# Microwave assisted extraction of mangiferin from *Curcuma amada*

**DOI:** 10.1007/s13205-011-0023-7

**Published:** 2011-09-10

**Authors:** K. Padmapriya, Abhishek Dutta, Surabhi Chaudhuri, Debjani Dutta

**Affiliations:** 1 Department of Biotechnology, National Institute of Technology, Durgapur, 713209 India; 2 BIOMATH, Department of Mathematical Modelling, Statistics and Bioinformatics, Ghent University, Coupure Links 653, 9000 Ghent, Belgium

**Keywords:** Mangiferin, Microwave power, Microwave irradiation time, Mathematical model

## Abstract

Mangiferin present in *Curcuma amada* was extracted with the help of microwave assisted extraction (MAE). The extraction solvent used was ethanol, which is eco-friendly and reduced the risk of environmental hazard. The mangiferin content was found to increase until 500 W, but decreased as the microwave power was increased further. A similar threshold was also obtained for microwave irradiation time. Following a mathematical analysis, an optimal mangiferin yield of 41 μg/mL was obtained from an extraction time of 15.32 s for a microwave power of 500 W.

## Introduction

Due to the presence of health promoting components such as vitamins, minerals, antioxidants and prebiotics, there is a growing interest in the extraction of the bioactive compounds from fruits, vegetables and spices. Several techniques are available for the extraction of bioactive compounds from plant sources including Soxhlet, microwave assisted extraction (MAE), ultrasound-assisted extraction (UAE), and heat reflux extraction (HRE). The main advantages of MAE are the considerable reduction in time and solvent as compared to the conventional techniques (Xiao et al. 2008). *Curcuma amada*, an understudied spice, belongs to the family Zingiberaceae, and has medicinal properties. It looks like ginger, but has mango flavour and is usually used for culinary purposes in the Indian sub-continent. Mangiferin is a phytochemical and is said to be present in mango ginger. It has a wide range of pharmacological activities including anti-diabetic, anti-HIV, anti-cancer, immune modulatory, anti-inflammatory and anti-oxidant properties (Wauthoz et al. 2007). However, no reports on MAE of mangiferin from *C. amada* have yet been published. In this study, we report on the feasibility of MAE for the extraction of mangiferin from *C. amada.* Our work aims to determine the effect of different microwave power and irradiation time on the mangiferin extraction from *C. amada* along with the kinetic parameters for efficient extraction through simple mathematical approach.

## Materials and methods

Mango ginger (*C. amada*) was purchased from a local supplier in Durgapur, India. The rhizomes were washed, sliced and dried in a hot air oven (OVFU, India) at 60 °C for 72 h and powdered in a grinder. Mangiferin standard was purchased from Sigma Aldrich, USA. The extraction of mangiferin was carried out with the method of Krivut et al. (1985) with slight modifications. The *C. amada* extract obtained after microwave irradiation was evaporated in a Rotary Vacuum Evaporator (Yamato, Japan), filtered and separated to remove other inactive molecules using chloroform and butanol. The final purified extract was evaporated and dissolved in DMSO and analysed with a UV–vis spectrophotometer (UV-2310, Techcomp), at 410 nm. The estimation of mangiferin was followed using the method described by Joubert et al. 2008.

## Statistical analysis

The statistical software SPSS 11 (SPSS, Chicago, IL) was used for the data analysis. The two factors, microwave power (MP, main factor) and microwave irradiation time (MIT, random factor), were treated on a randomised complete block design. Means and coefficients of variance were computed for all qualitative analysis and treatments with homogeneous means ranked using the Newman–Keuls post hoc test. The effect of these two factors with mangiferin content was compared using the bi-variant correlation analysis. A paired sample *T* test was used to compare the differences between two groups. The significance of the results was established at values greater than 0.05 in all the experiments performed.

## Result and discussion

### Effect of microwave power and irradiation time on the mangiferin yield

**Fig. 1:**
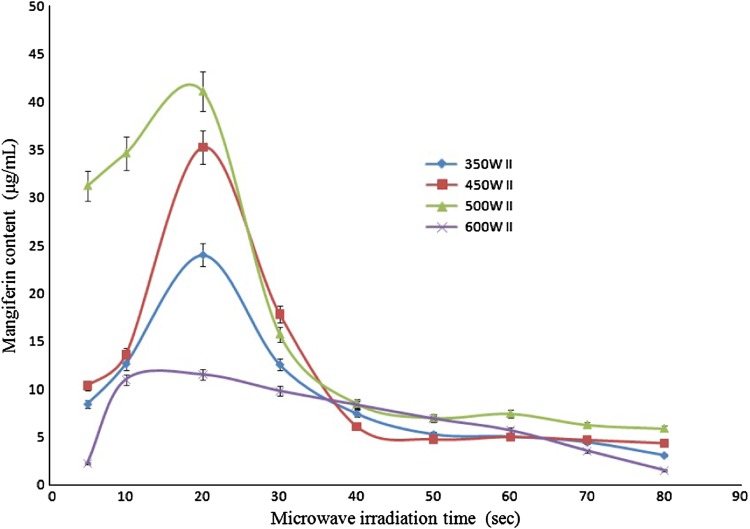
Effect of microwave power (350–600 W) and microwave irradiation time (5–80 s) on the mangiferin concentration

2.5 gm of dried powder of *C. amada* was extracted with 50 mL of 50% aqueous ethanol under different microwave power (350, 450, 500 and 600 W) for different MIT (5–80 s), as shown in Fig. 1. The solvent used was 50% ethanol, as the solvent of medium polarity was found to improve mangiferin extraction (unpublished results). The concentration of mangiferin and the extraction efficiency were found to improve when the microwave power was raised from 350 to 500 W. When the microwave power was raised from 350 to 500 W, electromagnetic energy was transferred to the extraction system quickly and this improved the extraction efficiency. However, the concentration of mangiferin decreased at 600 W microwave power. Similar results were observed by Xiao et al. (2008) in the extraction of flavonoid from *Radix astragali* at high power due to non-orderly molecular interactions. During shorter irradiation time (till 20 s), the mangiferin content increased with microwave power. However, when the extraction solution was heated beyond 20 s the mangiferin content reduced, and this reduction was observed in all microwave powers. This might be due to the high temperature involved in the extraction process which might have caused degradation of mangiferin (Kim et al. 2010). The mangiferin content was 24 μg/mL, 35.26 μg/mL and 41.10 μg/mL at 350 W, 450 W and 500 W, respectively, with an exposure of 20-s irradiation time which decreased to 3.083 μg/mL, 4.35 μg/mL and 5.87 μg/mL after 80 s of MIT. This result indicates that very high irradiation time is not suitable for mangiferin extraction.

Statistical results indicate that the mangiferin concentration is negatively correlated with both microwave power (insignificant, *p* = 0.721) and MIT (significant, *p* = 0.000). The paired sample *T* test is significant between the two groups, i.e. mangiferin concentration with MIT and microwave power (MP). The results of analysis of variance (ANOVA) are given in Table 1. Both microwave power and MIT along with their interaction is significantly different. Newman-Keuls test shows that the mangiferin content is significantly different at all microwave power, with microwave power of 500 W yielding the highest mean mangiferin content. In case of MIT, there is no significant difference in mangiferin content at 50 and 60 s. However, the mangiferin content differ significantly with remaining MIT. Statistical results indicate that the optimal mangiferin yield was obtained at 500 W microwave power and irradiation time of around 20 s.

**Table 1:** Results of a one-way ANOVA showing the significance of the two sources (MIT and MP) on the dependent variable (mangiferin concentration) (*R*^2^ = 1, adjusted *R*^2^ = 0.999)

Source	Degree of freedom	Type III sum of squares	Mean square	*F* value	Significance
Microwave power (MP)	1122.190	3	374.063	5269.203	0.000
Microwave irradiation time (MIT)	3990.157	8	498.770	7025.866	0.000
MP × MIT	1680.888	24	70.037	986.569	0.000
Error	2.556	36	7.099E−02		
Corrected total	6795.791	71			

### Mathematical model formulation

**Fig. 2:**
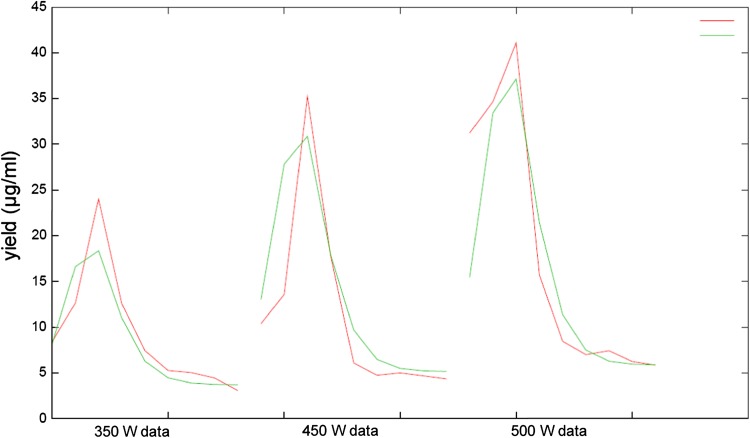
Proposed mathematical model (in *green*) for the microwave extraction procedure using the experimental data (in *red*) indicating optimal fit

Considering the two process parameters namely, the MIT and microwave power, an extraction model describing the relationship between these parameters and the extraction efficiency was constructed. The parameters of the model were fitted with a non-linear least-squares (NLLS) Marquardt–Levenberg algorithm, using the device-independent plotting program Gnuplot. It must be noted that the mean-averaged data used in the mathematical modelling is obtained by repeating the extraction process using different samples of *C. amada*. The response for 600 W was found to be different from the other three responses (i.e. from 350, 450 and 500 W) for all the samples studied, and did not follow a clear sequence. The same had been observed for the response at 600 W microwave power using statistical analysis. The dataset of 600 W was, therefore, neglected while validating the model. This assumption is further strengthened by the statistical analysis, mentioned in the previous section, that a probable optimum can be found at 500 W and somewhere around 20 s of irradiation time. Based on the experimental data (see Fig. 1), the following expression was formulated:1where *x* and *y* denotes irradiation time (in s) and microwave power (in W), respectively. Equation (1) was obtained by presuming one term constant in time (evolving a little with power since the time evolutions do not reach zero but to some positive limit), and the other term (which is the product of exponential decay in time with a certain power in time) modulated linearly by power. The optimal fit (in green) of the experimental data (in red) using the mathematical model is shown in Fig. 2. The optimal values of the parameters obtained due to regression are given in Table 2.

**Table 2:** Parameter values optimised using non-linear least-squares (NLSS) method

Parameters	Values	Asymptotic SE (±)
*a*_0_	0.106	0.1024
*a*_1_	217.723	92.32
*a*_2_	5.3754	1.59
*a*_3_	2.8665	0.8596
*a*_6_	69.222	109.5
*a*_7_	93.221	534.9

Using the mathematical model, an optimal yield of 40.65 μg/mL was obtained from an irradiation time of 15.32 s for a microwave power of 500 W. The root-mean-square (RMS) value of residuals and the variance of residuals (reduced Chi-square) was calculated to be 5.38 and 28.98 based on 21 degrees of freedom, respectively. Note that the tolerance value set for the parameter estimation is 1 × 10^−5^. The predicted model fitted well with the experimental data. It can be concluded that the proposed model correctly describes the MAE of mangiferin from *C. amada*. However, to obtain a more accurate fit, it is necessary to use additional process parameters for the mathematical model such as solvent mass ratio, solvent concentration, extraction cycle and extraction (leaching) time. These factors have been found to influence mangiferin yield while using traditional extraction techniques.

## Conclusion

This study demonstrates the feasibility of microwave assisted extraction of mangiferin from *C. amada*. Experimental results showed that the mangiferin content initially increased up to 20 s of irradiation time for all microwave power studied. A statistical analysis was performed which indicated an optimum mangiferin concentration at 500 W for an irradiation time of around 20 s. Optimal values of irradiation time and microwave power was obtained using a mathematical model based on nonlinear least-squares technique. Moreover, usage of ethanol as an eco-friendly alternative overcomes the serious hazards posed by various techniques adapted by humans. Thus, the extraction of mangiferin from *C. amada* using microwaves and ethanol is efficient not only from the industrial point of view by preventing environmental hazards, but also can be controlled using simple mathematical model. However, a rigorous experimental analysis using several influencing factors needs to be performed which can help posit the mathematical relationship justified by the mechanisms involved.
